# Endogenous Nociceptin / Orphanin FQ System Involvement in Hypothalamic-Pituitary-Adrenal Axis Responses: Relevance to Models of Inflammation

**DOI:** 10.1111/j.1365-2826.2009.01912.x

**Published:** 2009-11

**Authors:** J D Leggett, K L Dawe, D S Jessop, A J Fulford

**Affiliations:** * Department of Anatomy, School of Medical Sciences, University of BristolBristol, UK; † Henry Wellcome Laboratories for Integrative Neuroscience and Endocrinology, University of BristolBristol, UK

**Keywords:** nociceptin, nociceptin/orphanin FQ peptide receptor, hypothalamic pituitary adrenal axis, lipopolysaccharide, mRNA

## Abstract

Nociceptin/orphanin FQ (N/OFQ) peptide and its receptor (NOP) function in the neuromodulation of anxiety, stress and hypothalamic-pituitary-adrenal (HPA) axis activity. We investigated the endogenous NOP system using the selective NOP antagonist, UFP-101, during the HPA axis response to bacterial endotoxin, lipopolysaccharide (LPS). Although i.c.v. N/OFQ (1 μg/rat) had no significant effect on LPS-induced (250 μg/rat i.p) plasma corticosterone release at 30 or 60 min post-i.c.v. injection, i.c.v. UFP-101 (1 μg/rat)/LPS significantly attenuated plasma adrenocorticotrophic hormone and corticosterone at the 30-min time-point compared to i.c.v saline (0.9%)/LPS. Parvocellular paraventricular nucleus (PVN) corticotrophin-releasing factor (CRF) and corticotrophic pro-opiomelanocortin (POMC), but not parvocellular PVN arginine vasopressin (AVP), mRNA expression was significantly increased by LPS compared to non-LPS control. Intracerebroventricular UFP-101/LPS treatment was associated with increased POMC mRNA expression 4 h after injection and a clear trend towards increased parvocellular CRF mRNA. Furthermore, i.c.v. UFP-101 was selectively associated with an LPS-induced increase in parvocellular AVP mRNA, an effect that was absent in the i.c.v saline/LPS group. To determine whether LPS challenge was associated with compensatory changes in N/OFQ precursor or NOP receptor mRNAs, in a separate study, we undertook reverse transcriptase-polymerase chain reaction analysis of preproN/OFQ and NOP transcripts. In support of an endogenous role for central N/OFQ in inflammatory stress, we found that LPS significantly increased preproN/OFQ transcript expression in the hypothalamus 4 h after injection compared to the saline control. No changes in preproN/OFQ mRNA level in the hippocampus or basal forebrain (including bed nucleus of stria terminalis) were seen, albeit at 4 h. LPS was associated with a significant attenuation of NOP mRNA in the basal forebrain at 4 h, possibly as a compensatory response to increased N/OFQ release. Although the exact mechanisms require elucidation, the findings obtained in the present study provide evidence indicating that the endogenous NOP system is involved in the acute HPA axis response to immune challenge.

The peptide nociceptin/orphanin FQ (N/OFQ) is widely distributed throughout the central nervous system (1–3) and immune systems, consistent with its broad spectrum of actions. Its effects are mediated via activation of the specific G-protein-coupled receptor, NOP (1, 4–6). The N/OFQ-NOP system robustly modulates pain threshold, learning and memory, locomotion, cardiovascular parameters (7–9) and has significant potential with respect to neuroendocrine and neuroimmune integration. Various populations of immune cells, including mouse lymphocytes (10), human peripheral blood mononuclear cells (11) and circulating lymphocytes and monocytes (12, 13), express the NOP receptor. Our laboratory has recently demonstrated that a range of stimuli relevant to inflammation, including mitogens, corticotrophin-releasing factor (CRF) and pro-inflammatory cytokines, all increase endogenous N/OFQ secretion by immune cells in culture and that N/OFQ itself may subserve anti-inflammatory actions involving suppression of interleukin (IL)-2 (14) and tumour necrosis factor (TNF)-α secretion, with potential interaction with prostanoids (15).

In models of inflammation, exogenous N/OFQ application exerts multifunctional effects in neurones and glia, causing inhibition of lipopolysaccharide (LPS)-induced IL-1β, IL-6 and TNF-α release from cultured astrocytes (16), LPS-induced IL-1β mRNA expression in cerebral cortical microglia (17), attenuation of surgical-stress-induced macrophage IL-1β transcript expression (18) and down-regulation of spinal dorsal horn IL-1β, IL-6 and TNFα mRNA expression in adjuvant-induced arthritis (16). These findings are consistent with protective anti-inflammatory effects of N/OFQ over local and systemic immune responses including neuroinflammation. Furthermore, expression of N/OFQ-like immunoreactivity increases in embryonic and adult mouse dorsal root ganglion neurones after LPS treatment *in vitro* (19), LPS and pro-inflammatory cytokines increase N/OFQ precursor mRNA in astrocytes (20, 21) and the superantigen, staphylococcal enterotoxin A (SEA), increases expression of N/OFQ precursor mRNA levels in amygdala and hypothalamic extracts of mouse brain (22). These findings identify the counter-regulatory role of N/OFQ-NOP with regard to neuroimmune responses. Interestingly, N/OFQ precursor knockout mice show attenuated lymphoid organ expression of cytokine transcripts (TNFα and IL-1β) after challenge with SEA, and N/OFQ peptide given systemically to normal mice stimulates levels of circulating TNFα in response to SEA (23), which is consistent with *in vivo* immunomodulatory actions. Thus, the theoretical potential of the N/OFQ-NOP system with regard to inflammatory disorders or conditions associated with neuroimmune activation, including neurodegenerative or neuropsychiatric diseases, necessitates further evaluation, especially in consideration of the fact that N/OFQ precursor knockout mice display elevated basal and post-stress corticosterone levels compared to wild-type mice (24), which complicates the assessment of immunological responsiveness *in vivo*.

It is important to consider the central mechanism of action of NOP receptors in the control of neuroinflammation. Although recent research indicates a role for N/OFQ-NOP in the peripheral immune system, little is known regarding the central role of N/OFQ-NOP in inflammation models *in vivo*. In particular, we sought to investigate the interaction between N/OFQ-NOP and hypothalamic-pituitary-adrenal (HPA) axis activity, which is known to be critical to the inflammatory response, on the basis of recent evidence supporting a neuroendocrine role for N/OFQ-NOP. Stress studies have concentrated on the role of N/OFQ-NOP in models relevant for psychological stress (25, 26). For example, central injection of N/OFQ peptide augments the corticosterone response to novel environment stress in rats but has no effect on restraint stress-induced corticosterone levels (27). We have demonstrated that central injection of the peptidic NOP antagonist, UFP-101, ([Nphe^1^,Arg^14^,Lys^15^]N/OFQ-NH_2_), is able to fully attenuate the effects of i.c.v. N/OFQ on HPA axis activity (28) and prolong restraint stress-induced corticosterone release (29). This suggests that endogenous N/OFQ neurotransmission, and concomitant activation of the NOP receptor, are important for restraining the HPA axis response to the physical/psychological stress of restraint. Because different types of stressor activate the HPA axis, potentially invoking hierarchical pathways, the effect of N/OFQ-NOP system involvement in the HPA axis response to other models of stress [e.g. physical/immunological challenge using bacterial endotoxin (LPS)] is required to establish the functional activation of central N/OFQergic pathways and to clarify the role of N/OFQ-NOP in the fine-tuning of the acute stress response.

We have previously shown that i.c.v. UFP-101 is able to prolong the acute HPA axis response to a 60-min restraint stress (29), implicating endogenous N/OFQ release in the termination of the neuroendocrine stress response. In the present study, the principal aim was to determine the effects of UFP-101 on the HPA response to LPS challenge (i.e. an immunological stressor). In addition, we also characterised the effect of i.c.v. N/OFQ on the corticosterone response to LPS challenge and the effect of systemic LPS on prepro-nociceptin (preproN/OFQ) and NOP mRNA levels in the rat forebrain.

## Materials and methods

### Animals

Adult male Sprague-Dawley rats weighing 200–220 g (Harlan, Bicester, UK) were housed in a temperature and humidity controlled environment under a 12 : 12 h light/dark cycle (lights on 07.00h). Food and water were available *ad lib* and all experiments were carried out in strict accordance with UK. Home Office regulations [Animals (Scientific Procedures) Act 1986].

### Surgery

Three days prior to experiment rats were implanted with an intravenous (i.v.) cannula placed in the right external jugular vein under Saffan anaesthesia (1.4 ml/kg; Schering-Plough, Welwyn Garden City, UK). Anaesthesia was maintained via the i.v. cannula with a stainless steel tubing i.c.v. guide cannula (22-gauge) terminating 1 mm above the left lateral ventricle [0.8 mm caudal and 1.5 mm lateral to Bregma; coordinates derived from the atlas of Paxinos and Watson (30)] implanted and fastened to the skull with micro-screws and dental cement. A dummy cannula was inserted into the guide cannula to prevent any blockage during the recovery period and was removed the evening prior to the experiment so that animals would remain unstressed on the morning of the experiment. Guide and dummy cannulae were obtained from Plastics One (Roanoke, VA, USA). After surgery, rats were individually housed. During the post-operative period, the i.v. cannulae were flushed daily with heparin (25 IU/ml) via a sampling line to maintain patency and habituate the rats to the experimental procedure.

### Experiment 1: Effect of nociceptin i.c.v. on the corticosterone response to LPS

All experiments began between 09.30–10.00 h. Animals were randomly assigned into four treatment groups and received i.c.v. injections (5 μl total volume in 1 min) of either vehicle [0.9% sterile saline (Sal)] or N/OFQ (1.0 μg/rat in 0.9% sterile saline; Tocris, Bristol, UK). Immediately after i.c.v. injection, rats received i.p. injections of either lipopolysaccharide (250 μg/0.5 ml 0.9% sterile saline; Sigma, Poole, UK, LPS serotype 055:B5) or 0.9% sterile saline control (0.5 ml). Treatment groups comprised Sal/Sal control, Sal/LPS, NOFQ/Sal or NOFQ/LPS. Blood samples (250 μl) for the determination of basal plasma corticosterone concentrations were taken prior to the administration of i.c.v. and i.p. injections. Further blood samples were taken at 30 and 60 min post-i.c.v. injection. All blood sampling was carried out with the animal freely moving about the home cage via a heparinised saline-(25 IU/ml) filled sampling line connected to the previously implanted i.v. cannula. After each blood sample was taken, the volume of blood removed was replaced with an equal volume of heparinised saline. Plasma was obtained from blood samples by centrifugation and the samples stored at −20 °C until assayed.

### Experiment 2: Effect of the NOP antagonist, UFP-101, i.c.v. on the HPA axis response to LPS

In this study, animals were randomly assigned to four treatment groups. Two of the groups received i.p. injections of 0.9% sterile saline (0.5 ml total volume) immediately after an i.c.v injection of 0.9% sterile saline control (5 μl total volume injected over 1 min) or UFP-101 (1 μg/rat in 5 μl sterile 0.9% saline vehicle injected over 1 min). These represented the non-LPS groups. The remaining two groups of rats received i.p. injections of LPS (250 μg in 0.5 ml sterile 0.9% saline; as above) immediately after i.c.v. injection of sterile 0.9% saline (5 μl total volume) or UFP-101 (1 μg/rat in 5 μl sterile 0.9% saline). Treatment groups comprised Sal/Sal control, UFP/Sal, Sal/LPS or UFP/LPS.

Blood samples (250 μl) for the determination of basal plasma hormone concentrations were taken prior to the administration of i.c.v. and i.p. injections. Further blood samples were taken at 30 and 60 min post-i.c.v. injection. All blood sampling was carried out with the animal freely moving about the home cage via a heparinised saline-filled (25 IU/ml) sampling line connected to the previously implanted i.v. cannula. After each blood sample was taken, the volume of blood removed was replaced with an equal volume of heparinised saline. Plasma was obtained from blood samples by centrifugation and the samples stored at −20 °C until assayed. At 4 h post-i.c.v. injection, animals were killed by decapitation and the brains and pituitaries dissected out, frozen on dry ice and stored at −80 °C. Sections (12 μm thick) of pituitary and brain (including the hypothalamic paraventricular nucleus, PVN) were prepared using a cryostat (Bright Instruments, Huntingdon, UK) and stored at −80 °C until required for *in situ* hybridisation.

### Determination of plasma hormone concentrations

Plasma adrenocorticotrophic hormone (ACTH) concentrations were determined using a commercially available enzyme-linked immunosorbent assay (ELISA) kit (Biomerica, California, CA, USA). Plasma samples were diluted 1 : 5 in Biomerica CAL A zero calibrator buffer (bovine serum albumin/equine serum solution). The reported detection limit was 0.46 pg/ml with a calculated intra-assay variation of < 5% and an inter-assay variation of < 12%. Plasma corticosterone concentrations were determined as described previously (Harbuz *et al.* 1992) using antiserum supplied by Dr G. Makara (Institute of Experimental Medicine, Budapest, Hungary). The tracer used was [^125^I]-corticosterone (MP Biomedicals, Cambridge, UK) with a specific activity of 2–3 mCi/mg. The intra-assay coefficient of variation was < 12%.

### *In situ* hybridisation histochemistry

*In situ* hybridisation histochemistry was performed as described previously by Harbuz and Lightman (31). Oligonucleotide probes complementary to part of the exonic sequences of arginine vasopressin (AVP), CRF or pro-opiomelanocortin (POMC) mRNA and previously characterised (31, 32) were used. Probes were labelled with [^35^S]-labelled dATP (1250 Ci/mmol; Perkin Elmer, Boston, MA, USA) using terminal deoxyribonucleotidyl transferase (Roche, Burgess Hill, Sussex, UK) and column purified with Nuctrap Purification Columns (Stratagene, La Jolla, CA, USA). Approximately 100 000 c.p.m. of probe per 45 μl of hybridisation buffer was applied to each slide. Hybridisation was performed overnight at 37 °C. All the sections for each experiment were processed at the same time. Sections were then washed at 55 °C and room temperature in sodium citrate buffer and rinsed in distilled water before being air dried. Dried sections, together with a [^14^C]-labelled standard (American Radiolabeled Chemicals Inc., St Louis, MO, USA) were opposed to Hyperfilm autoradiography film (Amersham Biosciences, Little Chalfont, UK). AVP- and POMC-labelled sections were exposed for 3 days and CRF-labelled sections were exposed for 14 days. After film development, densitometric analysis of the autoradiographic images was carried out using a computer-assisted image analysis system (nih image, version 1.62; NIH, Bethesda, MD, USA). Optical density values were referenced against the linear portion of the calibration curve produced using the [^14^C] standards. Analysis of parvocellular AVP mRNA level involved filtering out the signal from the magnocellular component of the PVN using the thresholding tool available in NIH image. Because magnocellular neurones contain a large excess of AVP mRNA compared to the parvocellular cells and therefore bind more probe, the darker signal can be readily identified on the film and omitted from the analysis. Data are presented as the mean ± SEM percentage change from control (assigned an arbitrary value of 100%) . Although we are confident of the robustness of this technique and its ability to identify subtle changes in parvocellular AVP mRNA (33), this method of analysis may increase the possibility of false negative results.

### Experiment 3: Effect of LPS treatment on preproN/OFQ and NOP receptor mRNA level in rat forebrain

#### Brain dissection

Animals were administered LPS i.p. (250 μg/rat) or sterile saline (0.9%) control (in a volume of 0.5 ml/rat) 4 h prior to killing and removal of the brain. This time-point was selected on the basis of previous studies showing SEA-induced changes in preproN/OFQ expression in the brain (22). Brains were subsequently dissected according to the method of Glowinski and Iversen (34), as described previously (35), carried out under sterile conditions on pre-chilled Petri dishes. The cerebellum and pons were separated from the rest of the brain and a transverse section made at the level of the optic chiasm that defines the anterior part of the hypothalamus and passes through the anterior commissure. The hypothalamus, basal forebrain [comprising the caudate nucleus, nucleus accumbens, globus pallidus, ventral pallidum and bed nucleus of stria terminalis (BNST)] and hippocampus regions were excised and subject to the RNA extraction procedure.

#### Isolation of RNA from rat brain

RNA was isolated from rat brain tissue (75-mg samples) by guanidinium thiocyanate–phenol–chloroform extraction according to the method of Chomczynski and Sacchi (36). After dissection, brain samples were immediately placed into 750 μl of lysis buffer (4 m guanidinium thiocyanate (denaturing solution), 25 mm sodium citrate, 0.5% sarcosyl, 0.1 mβ-mercaptoethanol). Lysed cell products were transferred to a sterile microcentrifuge tube to which 75 μl of sodium acetate (2 m, pH 4.0), 750 μl of water-saturated phenol and 150 μl of chloroform : isoamyl alcohol (49 : 1) were added. Samples were then shaken for 10 s to form an emulsion and then cooled on ice for 15 min followed by centrifugation at 4 °C for 20 min at 10 000 ***g***. The aqueous upper phase was transferred to a clean microcentrifuge tube and mixed with an equal volume of isopropanol at −20 °C for at least 1 h to enable RNA precipitation. After centrifugation at 4 °C for 20 min at 10 000 ***g***, the supernatant was discarded and the RNA pellet resuspended in guanidinium thiocyanate denaturing solution. RNA was reprecipitated by addition of an equal volume of isopropranol at –20 °C for a further 1 h. After centrifugation at 4 °C for 10 min at 10 000 ***g***, the RNA pellet was washed in 1 ml of cold 75% ethanol followed by a final centrifugation at 4 °C for 10 min at 7500 ***g***. The supernatant was discarded and the RNA pellet air-dried and resuspended in RNAse-free H_2_O. RNA concentration was determined by ultraviolet spectrophotometry using GeneQuant Pro (Amersham Biosciences) and purity was determined by calculation of the A_260/280 nm_ ratios. Total RNA samples (in 20 μl pure H_2_O) were then stored at −80 °C prior to reverse transcriptase-polymerase chain reaction (RT-PCR).

### RT-PCR for preproN/OFQ and NOP mRNAs

Levels of mRNA for preproN/OFQ, NOP receptor and GAPDH (i.e. house-keeping gene) were determined by semi-quantitative RT-PCR using the Expand Reverse Transcriptase kit (Roche). One microgram of total RNA and 1 μl Oligo(dT)_15_ (Roche) underwent initial denaturation for 10 min at 65 °C in a volume of 10.5 μl of RNAse-free H_2_O and were then immediately cooled on ice. Reverse transcription was then performed at 42 °C for 1 h by addition of 1× Expand Reverse Transcriptase Buffer, 10 mm dithiothreitol, 1 mm each deoxynucleotide triphosphate (dNTP), 20 U of ribonuclease inhibitor (RNAsin; Promega, Southampton, UK), and 50 U of Expand Reverse Transcriptase in a total volume of 20 μl. The reaction was terminated by placing sample tubes on ice. Primers for amplification of the cDNA for preproN/OFQ were: sense, 5′-CCCACGGCTGCACCATGAAAATC-3′; antisense, 5′-TTCCTCTCACCTGGCCCTACGAGA-3′ (37) with a predicted PCR amplification product of 840 bp. Primers for the cDNA for NOP receptor were: sense, 5′-ATGGAGTCCCTCTTTCCTGCT-3′; antisense, 5′-ACATGTTGTAGTAGTCGATAGCA-3′ with a predicted PCR amplification product of 399 bp. Primers for the cDNA for GAPDH were: sense, 5′-GAACGGGAAGCTCACTGGCAT-3′; antisense, 5′-GTCCACCACCCTGTTGCTGTTAG-3′ with a predicted PCR amplification product of 308 bp. NOP and GAPDH primer pairs were designed and validated in-house. cDNA aliquots (2 μl) were amplified by PCR in a 25-μl reaction volume by Taq DNA polymerase (0.6 U; Abgene, Epsom, UK) in reaction buffer [75 mm Tris–HCl pH 8.8, 20 mm (NH_4_)_2_SO_4_, 0.1% Tween 20] including 2 mm MgCl_2_, dNTPs (0.2 mm each) and 50 pmol of forward and reverse oligonucleotide primers. Amplification reactions were performed using a thermal cycler (Perkin-Elmer) and terminated prior to the end of the exponential phase (preproN/OFQ, 26 cycles; NOP receptor, 26 cycles; GAPDH, 20 cycles). Optimised amplification conditions for PCR involved denaturation at 95 °C for 2 min, followed by the appropriate number of cycles at 95 °C for 20 s, annealing at 55 °C for 45 s, extension at 72 °C for 1 min and completion by a final extension at 72 °C for 10 min. Amplified PCR products (15 μl) were mixed with 5 μl of loading buffer (0.5% sodium dodecyl sulphate, 25 mm ethylenediaminetetraacetic acid, 25% Ficoll, 0.15% bromophenol blue) and resolved on a 2% agarose gel containing 0.1 μg/ml ethidium bromide by electrophoresis. Semi-quantitative analysis of resolved products was achieved by visualisation and scanning densitometric measurement of band intensity using Bio-Rad Quantity One analysis software and the Gel Doc 2000 Gel Documentation System (Bio-Rad, Hercules, CA, USA). The relative band intensity (pixel intensity per band) of the PCR product (preproN/OFQ or NOP receptor) was expressed as a ratio against data for the house-keeping gene, GAPDH, determined in the same sample.

### Statistical analysis

Radioimmunoassay and ELISA data were analysed using two-way anova followed by the post-hoc Fisher protected least-significant difference test. Data from the *in situ* hybridisation experiments were analysed using one-way anova and Bonferroni post-hoc tests. For RT-PCR studies, Mann–Whitney U-tests were used to compare ratios of the mRNA of interest to GAPDH between groups. In all tests, P < 0.05 was considered statistically significant.

## Results

### Experiment 1: Effect of the N/OFQ peptide i.c.v. on the corticosterone response to LPS

**Fig. 1:**
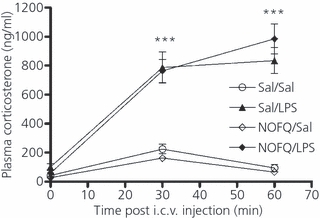
Plasma corticosterone concentration after treatment with i.c.v. saline or 1 μg/rat of nociceptin/orphanin FQ (N/OFQ) peptide immediately prior to i.p. injection of saline (Sal) or lipopolysaccharide (LPS, 250 μg/rat). Data are presented as the mean ± SEM plasma corticosterone concentration (ng/ml) , n = 7; ***P < 0.001 versus saline-treated groups.

Two-way anova indicated a significant effect of time (F_3,56_ = 58.4, P < 0.001) and treatment (F_3,56_ = 14.7, P < 0.001) on plasma corticosterone concentration (Fig. 1). LPS injection significantly increased plasma corticosterone release at 30 and 60 min post-injection in both i.c.v. injection groups, although N/OFQ peptide did not significantly modulate the LPS response. Saline injection i.p. to control rats was without significant effect on plasma corticosterone release at the 30- and 60-min time-point. There was also no significant effect of either saline or N/OFQ peptide i.c.v on plasma corticosterone in i.p. saline-injected rats.

### Experiment 2: Effect of the NOP antagonist, UFP-101, i.c.v. on the HPA axis response to LPS

#### Plasma hormone levels

**Fig. 2:**
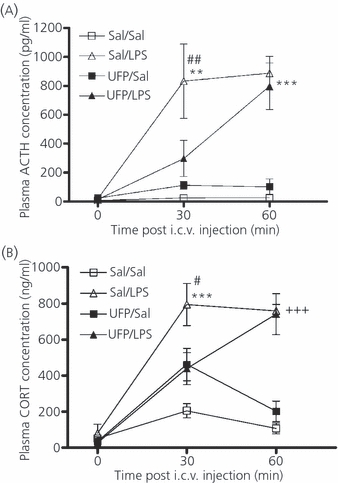
(a) Plasma adrenocorticotrophic hormone (ACTH) concentration after i.c.v. injection of saline or 1 μg/rat of UFP-101 (UFP) immediately prior to i.p. injection of saline (Sal) or 250 μg of lipopolysaccharide (LPS). Data are presented as mean plasma ACTH concentration (pg/ml) ± SEM for n = 5–10 (***P < 0.001 or **P < 0.01 versus respective saline i.p treatment; ##P < 0.01 versus UFP/LPS). UFP/LPS treatment was not significantly different to the UFP/Sal group. (b) Plasma corticosterone (CORT) concentration after i.c.v. injection of saline or 1 μg/rat of UFP-101 (UFP) immediately prior to i.p. injection of saline (Sal) or 250 μg of LPS. Data are presented as the mean ± SEM plasma corticosterone concentration (ng/ml), n = 5–9; ***P < 0.001 versus Sal/Sal group; +++P < 0.001 versus respective saline i.p. treatment; #P < 0.05 versus UFP/Sal and UFP/LPS groups.

Two-way anova indicated a significant effect of time (F_3,48_ = 19.87, P < 0.001) and treatment (F_3,48_ = 24.72, P < 0.001) on plasma ACTH concentration (Fig. 2a). There were no significant differences in basal levels of plasma ACTH at the 0 time point. There were no significant differences between the i.p. saline-treated control groups (Sal/Sal and UFP/Sal) at any time point throughout the experiment. Although LPS treatment caused a significant increase in plasma ACTH release, there was a significant difference between Sal/LPS and UFP/LPS groups at the 30-min time-point. Sal/LPS significantly increased plasma ACTH compared to the respective Sal/Sal control group at 30 min post-i.c.v. injection (Sal/Sal: 24.9 ± 6.63 pg/ml, n = 9; Sal/LPS: 833.0 ± 257.2 pg/ml, n = 5, P < 0.001). UFP/Sal treatment had no significant effect on ACTH secretion at 30 min post-injection (UFP/Sal: 111.6 26.9 pg/ml, n = 6); however, UFP-101 significantly attenuated the effect of LPS on plasma ACTH level at 30 min (UFP/LPS: 297.8 ± 125.7 pg/ml, n = 8, P < 0.01). At 60 min post-i.c.v. injection, the LPS-induced plasma ACTH response was elevated to an equivalent extent between i.c.v saline and i.c.v. UFP-101 groups (Sal/LPS: 888.5 ± 116.4 pg/ml, n = 5; UFP/LPS: 798.0 ± 160.7 pg/ml, n = 8, P < 0.001) compared to their respective i.p. saline groups (Sal/Sal: 26.2 ± 15.96 pg/ml, n = 9; UFP/Sal: 101.9 ± 54.83, n = 6).

Two-way anova also indicated a significant effect of time (F_3,50_ = 54.15, P < 0.001) and treatment (F_3,50_ = 17.34, P < 0.001) on plasma corticosterone secretion (Fig. 2b). There was no significant difference in the basal levels of corticosterone at time point 0. Post-hoc tests indicated a significant difference at the 30-min time point between the two groups that received saline i.p. injections (Sal/Sal and UFP/Sal), indicating that, under the UFP/Sal condition, plasma corticosterone was increased compared to the Sal/Sal control (P < 0.05). Consistent with the ACTH results i.p. LPS treatment significantly increased plasma corticosterone concentration at 30 min in the Sal/LPS group compared to the i.p. saline control (Sal/Sal: 205.3 ± 40.08 ng/ml, n = 9; Sal/LPS: 795.6 ± 117.6 ng/ml, n = 5, P < 0.001). Furthermore, i.c.v. UFP-101 significantly attenuated the LPS-induced CORT increase at the 30-min time-point (P < 0.05), such that there was no difference between UFP/Sal and UFP/LPS groups at this time (UFP/Sal: 461.9 ± 90.35 ng/ml, n = 7; UFP/LPS: 439.7 ± 88.19 ng/ml, n = 8). However, at 60 min post-i.c.v injection, plasma corticosterone levels were significantly elevated to an equivalent level in both LPS-treated groups compared to the i.p. saline control groups (Sal/LPS: 759.2 ± 35.22 ng/ml, n = 5; UFP/LPS: 741.5 ± 113.8 pg/ml, n = 8, P < 0.001).

#### mRNA expression in the PVN and pituitary

**Fig. 3:**
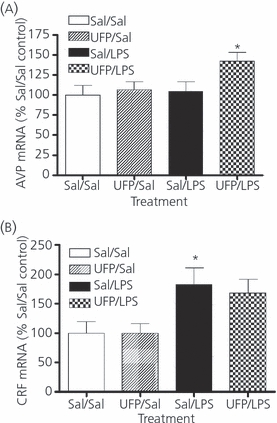
Arginine vasopressin (AVP) and corticotrophin-releasing factor (CRF) mRNA expression (a, b) in the parvocellular region of the hypothalamic paraventricular nucleus 4 h after treatment with i.c.v. saline or 1 μg/rat UFP-101 (UFP) immediately prior to i.p. saline (SAL) or 250 μg of lipopolysaccharide (LPS). Data are presented as the percentage of saline control ± SEM, n = 6–9, *P < 0.05.

**Fig. 4:**
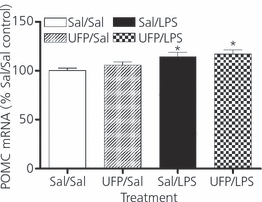
Pro-opiomelanocortin (POMC) mRNA expression in the anterior pituitary 4 h after treatment with i.c.v. saline or 1 μg/rat of UFP-101 (UFP) immediately prior to i.p. saline (SAL) or 250 μg of lipopolysaccharide (LPS). Data are presented as the percentage of saline control ± SEM, n = 7–9, *P < 0.05.

One-way anova indicated a significant difference in AVP mRNA expression between treatment groups (F_3,29_) = 3.020, P = 0.048; Fig. 3a). Post-hoc tests revealed a significant increase in AVP mRNA expression in the UFP/LPS group only compared to the control group (Sal/Sal: 100.0 ± 12.01%, n = 8; UFP/Sal: 106.7 ± 10.12%, not significant, n = 8; Sal/LPS: 104.7 ± 11.55%, not significant, n = 6; UFP/LPS: 142.0 ± 11.48%, P < 0.05, n = 8). Also in the hypothalamic PVN, one-way anova showed a significant difference in CRF expression between the treatment groups (F_3,30_ = 3.714, P = 0.023; Fig. 3b). Post-hoc tests revealed a significant increase in CRF expression induced by only the Sal/LPS group because the trend towards an increase seen with the UFP/LPS group just failed to reach significance (Sal/Sal: 100.0 ± 19.84%, n = 9; Sal/Sal: 99.49 ± 16.67%, not significant, n = 7; Sal/LPS: 182.5 ± 28.57%, P < 0.05, n = 7; UFP/LPS: 167.7 ± 24.20%, not significant, n = 8). In the pituitary, POMC mRNA expression was significantly affected by treatment (F_3,32_ = 4.605, P = 0.009; Fig. 4). POMC mRNA was significantly increased by both of the LPS groups only, post-hoc tests revealed no other significant differences between the groups (Sal/Sal: 100.0 ± 2.728%, n = 10; UFP/Sal: 105.5 ± 3.165%, not significant, n = 8; Sal/LPS: 114.1 ± 4.660%, P < 0.05, n = 7; UFP/LPS: 116.8 ± 4.516%, P < 0.05, n = 8).

### Experiment 3: Effect of LPS treatment on preproN/OFQ and NOP receptor mRNA expression in rat forebrain

**Fig. 5:**
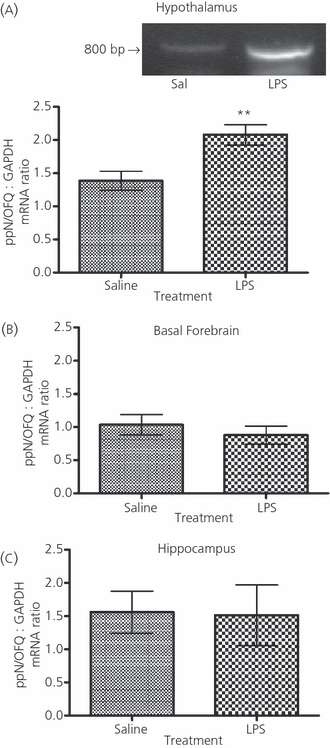
Effect of sterile saline (0.9%) or lipopolysaccharide (LPS) (250 μg/rat) on prepro-nociceptin/orphanin FQ (ppNOFQ) mRNA expression in rat (a) hypothalamus, (b) basal forebrain and (c) hippocampus 4 h post-injection. Data are expressed as ppNOFQ : GAPDH ratios (mean ± SEM, n = 6). **P < 0.01, Mann–Whitney U-test.

Levels of preproN/OFQ mRNA were examined by RT-PCR in hypothalamus, basal forebrain and hippocampus from rat brain. A representative digitised image showing ethidium bromide staining of cDNAs for preproN/OFQ in rat hypothalamus from saline control and LPS-treated rats is shown in Fig. 5. Band intensities provide an index of levels of the corresponding mRNAs in the rat hypothalamus. Statistical analysis revealed that LPS treatment significantly increased preproN/OFQ mRNA in the hypothalamus compared to saline-treated rats. There was a significant difference between the preproN/OFQ : GAPDH ratio in the hypothalamus in the LPS group, representing a 49.64% increase in preproN/OFQ mRNA (mean preproN/OFQ : GAPDH ratio = 2.08) relative to the control group (preproN/OFQ : GAPDH ratio = 1.39, n = 6 per group, Mann–Whitney U = 3.00, P < 0.01; Fig. 5a). LPS treatment, however, had no significant effect on preproN/OFQ mRNA levels in the basal forebrain or hippocampus 4 h post-injection (Fig. 5b, c).

**Fig. 6:**
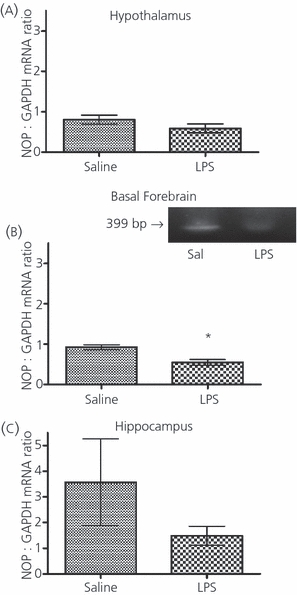
Effect of sterile saline (0.9%) or lipopolysaccharide (LPS) (250 μg/rat) on NOP mRNA expression in rat (a) hypothalamus, (b) basal forebrain and (c) hippocampus 4 h post-injection. Data are expressed as NOP : GAPDH ratios (mean SEM, n = 6). *P < 0.05, Mann–Whitney U-test.

Levels of NOP mRNA were detected by RT-PCR in hypothalamus, basal forebrain and hippocampus of LPS and saline-treated rats. A representative digitised image showing ethidium bromide staining of cDNAs for NOP mRNA in rat basal forebrain from saline control and LPS-treated rats is shown in Fig. 6. Statistical analysis revealed that there was a significant difference in the NOP : GAPDH ratio in the basal forebrain in LPS-injected rats (Control, mean ratio = 0.92; LPS, mean ratio = 0.55), P < 0.05; n = 6 per group; Fig. 6b), representing a 40.2% decrease in NOP mRNA in this region in the LPS group. LPS (250 μg/rat), however, had no significant effect on NOP mRNA level in hypothalamus or hippocampus 4 h post-injection (Fig. 6a, c).

## Discussion

In the present study, we have shown for the first time that the HPA axis activation response to bacterial endotoxin, LPS, challenge can be significantly attenuated by central blockade of NOP receptors using the NOP receptor antagonist UFP-101. The robust reduction in both plasma ACTH and corticosterone in response to LPS has important considerations for understanding the cooperative neuroimmune role of the N/OFQ-NOP system *in vivo*. The significant attenuation of LPS-induced ACTH and corticosterone release 30 min after i.c.v. infusion of UFP-101 indicates that the acute release of pituitary ACTH in response to LPS challenge is mediated via a pathway involving endogenous N/OFQ release and activation of NOP receptors. These results notably contrast with our recent findings showing that UFP-101 prolongs the corticosterone response to the stress of restraint (60 min), implicating endogenous N/OFQ function in the termination of the stress response (29). In the present study, the finding that UFP-101 attenuates the HPA axis hormone response to acute LPS injection suggests that central endogenous N/OFQ release is enhanced in response to inflammatory stimulation. However, enhanced release of N/OFQ and associated activation of the HPA axis resulting in enhanced production of adrenal glucocorticoids would effectively damp down the potentially damaging inflammatory response, exerting a N/OFQ protective effect *in vivo*. Interestingly, despite the clear attenuation of ACTH and corticosterone responses to LPS in the i.c.v. UFP-101-treated rats, when given alone, N/OFQ peptide i.c.v. did not extend the HPA axis activation. The dose of N/OFQ injected (1 μg/rat in 5 μl i.c.v.) is the same dose that we have previously demonstrated to activate the HPA axis under basal conditions (28), possibly implying a ceiling effect of the peptide in the LPS condition employed here. However, in the present study, we did not observe an increase in basal corticosterone secretion in response to i.c.v. N/OFQ, as previously shown (28). It is entirely possible that the i.p. saline injection procedure and associated handling influenced the response to i.c.v. N/OFQ in the present study. Furthermore, full dose–response studies would be necessary to investigate the ability of exogenous N/OFQ peptide application to modulate the HPA axis response to LPS challenge *in vivo*. The saline i.p. injection may also, in part, explain the observation that i.c.v. UFP-101/saline i.p. treatment caused a short-lasting increase in corticosterone release, when, previously, we found no effect of i.c.v. UFP-101 infusion on basal HPA axis activity (28). Such findings indicate the potential of mildly stressful procedures to influence the endogenous N/OFQ tone *in vivo*.

Because the effect of UFP-101 in the present study was only observed in the first 30 min post-LPS injection, it is possible that it disrupts the mechanism or mechanisms responsible for the immediate, acute release of ACTH. By 60 min post-LPS administration, plasma ACTH and corticosterone levels in UFP-101-treated LPS rats were comparable with those of the LPS control rats and, by 4 h post-injection, CRF and POMC mRNA transcripts for both LPS-treated groups were elevated in response to activation of the HPA axis. The elevation of plasma ACTH and corticosterone at 60 min correlates with the reported time for peak peripheral LPS-induced plasma cytokine levels that contribute to the extended glucocorticoid response to immunological challenge (38–40). In response to LPS challenge, parvocellular PVN AVP mRNA expression was significantly elevated in the UFP-101-treated LPS rats versus the control 4 h after injection. Although LPS alone has been reported to elevate AVP mRNA expression and stimulate AVP release (39–41), this is not a consistent effect (42) and was also not observed in the present study. Centrally-injected N/OFQ has been reported to inhibit dehydration-induced AVP release and an inhibitory endogenous N/OFQ tone is involved in the regulation of plasma AVP concentration (43). It is therefore possible that the combination of LPS challenge and removal of endogenous N/OFQ tone by UFP-101 was able to stimulate up-regulation of parvocellular PVN AVP mRNA expression, presumably in response to enhanced AVP release at the median eminence.

In agreement with our *in vivo* findings showing UFP-101 mediated attenuation of the HPA axis hormone response to LPS, our RT-PCR studies confirmed that LPS is a potent regulator of N/OFQergic and NOP-responsive neurones in the brain. In addition to our recent *in vitro* research demonstrating LPS induction of immune-derived N/OFQ secretion, we have shown that the acute LPS model affects N/OFQ–NOP in terms of mRNA expression in the brain, consistent with a major role for this peptide in inflammation. Previous research has demonstrated that *in vivo* challenge models, such as carrageenan-induced paw inflammation, are associated with increased preproN/OFQ mRNA in dorsal root ganglia (DRG) (44, 45) and that LPS *in vitro* can also induce preproN/OFQ mRNA in DRG neurones (19). Consistent with the effects of UFP-101 i.c.v., supporting tonic regulation of HPA axis responses *in vivo*, our RT-PCR results suggest that, 4 h after systemic LPS injection, preproN/OFQ mRNA transcription is markedly increased in the hypothalamus. It is particularly interesting to note that, although a marked increase in synthesis of preproN/OFQ mRNA was seen in the hypothalamus post-LPS stress, no change in expression of NOP mRNA was detected in this region at the 4-h time-point. By contrast, NOP mRNA synthesis was significantly reduced in the basal forebrain region 4 h after LPS, without a change in preproN/OFQ mRNA transcription. Although it is difficult to interpret these findings in the context of local changes in gene expression after stress, it may be suggestive of dissociation between preproN/OFQ and NOP gene regulation, at least in terms of the response to LPS seen at this time-point. Interestingly, hippocampal preproN/OFQ and NOP mRNA transcript levels were not significantly affected by LPS, consistent with findings describing effects of SEA challenge on hippocampal expression of preproN/OFQ mRNA 1–4 h post-injection (22). SEA injection in mice was also reported to stimulate hypothalamic preproN/OFQ mRNA expression 2 and 4 h post-injection (22), which also concurs with our observations in rats.

Given our previous work implicating N/OFQ in the regulation of basal and stress-induced changes in HPA axis activity (28, 29), the LPS-induced up-regulation of preproN/OFQ synthesis is consistent with engagement of endogenous hypothalamic N/OFQ in HPA axis control. N/OFQ precursor mRNA is densely expressed in several medial hypothalamic subregions implicated in stress responses, including parvocellular paraventricular, arcuate, dorsal–medial and supramammillary nuclei (2). Further work is required to identify which preproN/OFQ-expressing neuronal sub-populations are activated after systemic LPS injection. Very few studies have addressed the effect of stress on regulation of forebrain preproN/OFQ expression; however it is interesting to note that food deprivation in rats, a probable stressor, does not alter hypothalamic expression of N/OFQ precursor mRNA across various discrete subregions (e.g. PVN, arcuate or ventromedial nuclei) (46).

The data obtained in the present study concerning the regulation of NOP mRNA synthesis after LPS are entirely novel and are of interest in the context of stress-induced changes in the central N/OFQ-NOP system *in vivo*. NOP receptor mRNA is widely distributed throughout the central nervous system, but is particularly densely expressed in hypothalamus and basal forebrain (3); therefore, an LPS-induced decrease in NOP mRNA level in the basal forebrain is of potential significance, although we cannot rule out the possibility that mRNA degradation may have influenced the NOP findings. In the present study, the basal forebrain region encompassed a number of structures, including the caudate putamen, globus pallidus and BNST, which express variable NOP mRNA density. Within the basal ganglia, the caudate putamen contains little NOP mRNA-expressing neurones compared to the globus pallidus, which is characterised by moderate density of NOP mRNA transcripts. By contrast, the BNST is notably rich in NOP mRNA, being especially high throughout the medial and posterolateral compartments (3). Taken together, this suggests that the moderate reduction in NOP mRNA after LPS could involve changes within the BNST. This hypothesis would be consistent with observations of LPS-induced neuronal activation of the PVN and dorsal BNST (47), in addition to LPS activation of BNST and PVN afferents (48). Efferent projections from hypothalamic nuclei such as PVN project to the BNST; therefore, it is also possible that the release of hypothalamic N/OFQ following LPS may correlate with the down-regulation of NOP receptors in forebrain projection sites, including the BNST.

Enhanced forward drive of the HPA axis after activation of immune-responsive brainstem neuronal populations and associated activation of N/OFQergic neurones may underpin compensatory changes in preproN/OFQ mRNA transcription. LPS may directly activate preproN/OFQ mRNA-expressing neurones because recent evidence reveals that LPS via binding to Toll-like receptor 4 (TLR4) complex directly activates DRG neurones (19). Furthermore, blocking TLR4 action with targeted antibodies prevents the LPS-induction of preproN/OFQ mRNA expression in DRG neurones, suggesting that highly homologous systems exist in peripheral neurones and immune cells for LPS binding and signal transduction. Whether such mechanisms occur in central neurones is not known; however, this would represent an entirely novel mechanism for direct LPS activation of N/OFQergic neurones.

## Conclusions

Administration of LPS is known to increase circulating pro-inflammatory IL-1β, IL-6 and TNFα, which drive a cascade of responses including HPA axis activation (39, 49, 50). IL-1β strongly upregulates N/OFQ secretion by immune cells (14), raising the possibility that pro-inflammatory cytokines drive up-regulation in preproN/OFQ mRNA in the hypothalamus. Although blockade of central NOP receptors was shown to be effective in attenuating an acute mechanism involved in the release of plasma ACTH observed up to 30 min, endogenous N/OFQ appears to have little involvement in the sustained hormonal response to LPS. The short-lasting inhibitory effect of UFP-101 on plasma hormone release may therefore be reflective of the truncated bioavailability of peptidic antagonists *in vivo*. The results obtained in the present study reinforce the importance of the endogenous N/OFQ-NOP system in the coordination of neuroendocrine and neuroimmune responses in inflammatory models.
